# Adaptations of an Effective Evidence-Based Pediatric Weight Management Intervention

**DOI:** 10.1007/s11121-023-01557-7

**Published:** 2023-07-21

**Authors:** R. T. Bartee, K. A. Heelan, C. A. Golden, J. L. Hill, G. C. Porter, B. A. Abbey, K. George, N. Foster, P. A. Estabrooks

**Affiliations:** 1 Department of Biology, University of Nebraska at Kearney, Kearney, NE USA; 2 Department of Kinesiology and Sport Sciences, University of Nebraska at Kearney, Kearney, NE USA; 3 School of Medicine, Population Health Sciences, University of Utah, Salt Lake City, UT USA; 4 Department of Health Promotion, University of Nebraska Medical Center, Omaha, NE USA; 5 Munroe-Meyer Institute, University of Nebraska Medical Center, Omaha, NE USA; 6 Department of Health and Kinesiology, University of Utah, Salt Lake City, UT USA

**Keywords:** Adaptations, Implementation outcomes, Child obesity

## Abstract

Current childhood obesity treatment programs do not address medically underserved populations or settings where all members of an interdisciplinary team may not exist—either within one organization or within the community. In this paper, we describe the use of a community-academic partnership to iteratively adapt Epstein’s Traffic Light Diet (TLD), into Building Healthy Families (BHF), a community-placed evidence-based pediatric weight management intervention (PWMI) and evaluate its effectiveness in reducing BMI *z* scores. Nine cohorts of families completed BHF. Participants included children aged 6–12 years with obesity (*M* = 9.46, SD = 1.74). The Framework for Reporting Adaptations and Modifications-Expanded guided our classification of modifications across BHF cohorts. Using the FRAME reporting structure, the changes that were documented were (1) planned and occurred pre-implementation, (2) based on decisions from local stakeholders (e.g., school administrator, members of the implementation team), and (3) specific to changes in content and context—with a focus on implementation and potential for local scale-up. The nature of the adaptations included adding elements (whole of family approach), removing elements (calorie counting), and substituting elements (steps for minutes of physical activity). Across 9 cohorts, 84 families initiated the BHF program, 69 families successfully completed the 12-week program, and 45 families returned for 6-month follow-up assessments. Results indicated that the BMI *z* score in children was reduced by 0.31 ± 0.17 at 6 months across all cohorts. Reduction in BMI *z* score ranged from 0.41 in cohort 4 to 0.13 in cohort 5. Iterative adaptations to BHF were completed to improve the fit of BHF to the setting and participants and have contributed to a sustained community PWMI that adheres to the underlying principles and core elements of other evidence-based PWMIs. Monitoring adaptations and related changes to outcomes can play a role in long-term sustainability and effectiveness.

## Introduction

A significant increase in childhood obesity since 1988 is well documented in national data (Fryar et al., 2020). This increase in prevalence of childhood obesity has resulted in a large body of literature and several systematic reviews that document the efficacy of pediatric weight management interventions (PWMIs) (Altman & Wilfley, 2015; Epstein et al., 1987; Golan & Crow, 2004; Reinehr, 2011; Reinehr et al., 2005; Wolfenden et al., 2010). Family-based interventions that target the parent, or the parent and child together, have efficaciously reduced and maintained child weight status (Golan, 2006; Golan & Crow, 2004; Golan & Weizman, 2001; Golan et al., 1998, 1999, 2006). The Traffic Light Diet (TLD) model developed by Epstein and colleagues first demonstrated efficacy ~ 40 years ago and included core principles with specific strategies for reducing caloric intake, increasing the intake of more healthful foods, decreasing the intake of less healthful foods, increasing physical activity, and building behavioral skills (Epstein, 1996; Epstein et al., 1986, 1990, 1994, 2007). Unfortunately, the degree to which efficacious PWMIs have been translated to, and are feasible in, typical rural or micropolitan areas is unclear (Findholt et al., 2013; Wilfley et al., 2017). Most efficacious PWMIs have been implemented and tested in clinical settings in larger cities, and current childhood obesity treatment recommendations do not address medically underserved populations or settings where all members of an interdisciplinary team may not exist—either within one organization or within the community (Wilfley et al., 2017). This is particularly significant when considering the prevalence of obesity is higher in micropolitan (i.e., towns with less than 50,000 residents) and rural areas when compared to urban areas (Joens-Matre et al., 2008). For families living in micropolitan and rural areas, community resources and the teams needed to implement PWMIs are often not available (Caldwell et al., 2016).

The body of literature related to PWMIs implemented in micropolitan and rural areas has demonstrated mixed results (Davis et al., 2013, 2016; Henes et al., 2010; Hill et al., 2019; Irby et al., 2012; Janicke, 2013; Janicke et al., 2008a; Ruebel et al., 2011; Smith & Holloman, 2013; Tripp et al., 2011). Thus, even though most of these PWMIs were developed from a common conceptual basis (i.e., physical activity promotion, healthful eating with portion or caloric reduction, lifestyle modification), they indicate being adapted from other efficacious approaches. Of the studies that explicitly stated that the implemented PWMI was adapted from an evidence-based approach (Hill et al., 2019; Janicke, 2013; Janicke et al., 2008b; Ruebel et al., 2011), the degree to which these studies specify the how, why, and what of adaptations is limited (Janicke, 2013; Janicke et al., 2008b).

Understanding evidence-based intervention adaptation is a scientific priority in dissemination and implementation research (Chambers & Norton, 2016; Rabin et al., 2018; Stirman et al., 2019). Within this context, adaptation is described as a process of thinking through and applying alterations in the delivery or design of an evidence-based intervention with an effort to improve local fit, effectiveness, or both (Stirman et al., 2019). Inherent in this description is that adaptations are planned and purposeful, which differs from other types of modifications that may be unplanned in reaction to unanticipated circumstances (Stirman et al., 2013). Adaptations can be considered from the perspective of the implementation team, intervention participants, or other stakeholder groups and can focus on content, context, or strategies to improve implementation and increase scalability (Stirman et al., 2019). Documenting and specifying adaptations to PWMIs and providing information on effectiveness can advance scientific knowledge in pursuit of broad adoption, implementation, and sustainability of PWMIs.

One approach that has shown promise for the successful adaptation of evidence-based interventions is the use of participatory approaches such as integrated research-practice partnerships, community-based participatory research, and community-academic partner initiatives (Estabrooks et al., 2019; Greenhalgh & Papoutsi, 2019; Hill et al., 2019). These approaches balance the focus on evidence-based intervention core components and local resources, values, and goals to ultimately achieve the best match between intervention characteristics and the local context. In this paper, we describe the use of an academic-community partnership to adapt Epstein’s TLD into a community-placed PWMI, Building Healthy Families (BHF), specify adaptations using the Framework for Reporting Adaptations and Modifications-Expanded (Stirman et al., 2019), and evaluate its effectiveness in reducing BMI *z* scores.

## Methods

### Study Context, Setting, and Participant Population

The adaptation of Epstein’s TLD, reported here, was initiated by a community-academic partnership that began with the associate superintendent of the local school district, school nurses, a community pediatrician, and academic partners with expertise in exercise and nutritional sciences. In 2005, this partnership successfully integrated a multicomponent body mass index (BMI) screening program with parental notification—the BMI Report Card—into the local public school system (Heelan et al., 2015). The partnership also identified a local gap in availability of PWMIs to support parents who received a BMI Report Card indicating that their child’s BMI percentile was ≥ 95 and therefore may be at risk for developing health risks. The community-academic partnership added a local clinical behavioral psychologist and a community dietitian, to adapt the TLD for local implementation as BHF. The implementation team consisted of the local behavioral psychologist, community dietitian, and physical education instructor at the host university. The program coordinator and other implementation staff were academic partners with expertise in exercise science.

The setting for these activities was a mid-western Micropolitan Statistical Area (population > 10,000 and < 50,000). During the course of the study, approximately 18.3% of households in the micropolitan area had children under the age of 18, and the median family income is $43,920 (U.S. Census Bureau, 2010). Approximately, 16–18% of the elementary school-aged children in this region had obesity. As with most micropolitan and rural communities, the resources for interdisciplinary PWMIs were limited. The implementation of BHF began as a community service of a small regional university that has historically focused on teacher training and undergraduate research.

Families were recruited throughout all nine cohorts using local newspaper advertisements, physician referrals, and BMI Report Cards through the public schools. Nine cohorts of families completed the BHF program between 2009 and 2016. Inclusion criterion included children aged 6–12 years old (*M* = 9.46, SD = 1.74) with a BMI percentile greater or equal to the 95th percentile for their age and gender. Child participants were mostly non-Hispanic white (84%) with slightly more than half (51%) female, which is representative of the population in this, and other, mid-west regions. The University of Nebraska at Kearney Institutional Review Board reviewed and approved the study (IRB No. 090408–1).

### Adaptation Framework and Process

We used the Framework for Reporting Adaptations and Modifications-Expanded (FRAME) (Stirman et al., 2019) to retrospectively describe our classification of modifications across BHF cohorts. This approach includes classifying adaptations based on (1) when and how in the implementation process the modification was made, (2) whether the modification was planned/proactive or unplanned/reactive, (3) who determined that the modification should be made, (4) what is modified (i.e., content, context, training and evaluation, or implementation and scale-up activities), (5) at what level of delivery the modification is made, (6) type or nature of context and content-level modifications, (7) the extent to which the modification is fidelity-consistent, and (8) the reasons for the modification, including (a) the intent or goal of the modification and (b) contextual factors that influenced the decision (Stirman et al., 2019). This approach is also characterized by the availability of coding tools to allow for specification and categorization of adaptations (Stirman et al., 2013).

The community-academic partnership that included school, clinical, and university members discussed initial adaptations of the TLD and used consensus decision-making processes (i.e., adaptations that were completed were discussed in detail and agreed to by all members). Following the initial adaptation, the implementation team, which included a balance of community (i.e., Registered Dietitian; Psychologist) and university partners (i.e., program coordinator; physical activity coordinator; evaluator), was responsible for adaptations made in subsequent cohorts. The consensus decision-making process was repeated with this group. The implementation team was responsible for adaptations made in subsequent cohorts. Implementation team members kept field notes and shared them weekly using informal check-ins. Implementation team members also met with a program evaluator after each weekly session to discuss factors influencing session delivery. After each completed cohort, the implementation team, program coordinator, and evaluator met as a group to consider data related to implementation outcomes (e.g., appropriateness, feasibility, and fidelity), including process evaluation data, member field notes, participant feedback, and participant data (e.g., attendance, attrition, most successful, least successful). The goal of this process was to modify the peripheral components of the TLD to maximize potential for effectiveness in the local setting while maintaining the integrity of the core elements of the intervention (Chambers & Norton, 2016).

### Individual Outcomes

#### Assessments

Families with at least one qualifying child who registered for the program were assessed at baseline, 12 weeks, and 6-month follow-up. Although all family members were encouraged to attend weekly sessions, only data on qualifying participants were analyzed (i.e., data from siblings that did not meet inclusion criteria were not included). Demographic information (age, race, ethnicity, sex) was collected at baseline during the informational meeting via family demographic form.

##### Anthropometric Measures

1. *Weight and height*. A calibrated digital Befour Platform Scale (model: PS6600) and Charder wall mounted digital stadiometer (model: HM-210D) were used to assess body weight and height, without shoes and in light clothing. All measurements were taken by two project staff who were trained for weight and height assessments.
2. *BMI and BMI z scores.* Height and weight, along with gender and age (in children), were used to compute BMI and BMI *z* scores using standard algorithms developed by the CDC (Kuczmarski et al., 2002).

### Analysis

Data analysis was undertaken using SAS software (version 9.4, SAS system for Windows). The purpose of this study was to report change in real-world, uncontrolled circumstances so missing values were not imputed in the primary analysis. However, we did assess imputation of baseline values in a secondary exploratory analysis, and differences in outcomes were non-significant (*p* > 0.05). Descriptive analyses were computed for child participants who entered the program, completed 12-week assessments, and those who returned for 6-month assessments. Although 6-month assessment data was collected, program “completers” were those who had baseline and 12-week assessment data available for analysis. The mean change in outcomes between pre- and post-12-week program were assessed using a linear mixed model. In addition, we determined differences between completers and non-completers for baseline measures.

## Results

### Adaptations Made for Cohort 1

Table 1 provides the key program components from Epstein’s Traffic Light Diet (Epstein et al., 2001) that were adapted for BHF pre-implementation by the community-academic partnership. All changes were made with the primary goal of making the program a better fit for local implementation in a micropolitan community while adhering to the underlying behavior modification, nutrition, and physical activity principles of the TLD. Using FRAME (Stirman et al., 2019) categories of adaptation for description, initial program adaptations for cohort 1 were mainly contextual (i.e., personnel, setting, format). Specifically, cohort 1 adaptations included interdisciplinary team implementation at a comprehensive undergraduate university, session duration of 90 min for 12 weekly sessions with maintenance up to 6 months, and a strong focus on whole family participation (i.e., both guardians and siblings). Examples of adaptations made for cohort 1 within the type or nature content construct include (1) BHF focused on red food goals and used a daily step goal for physical activity rather than a focus on energy intake and daily minutes of MVPA and (2) BHF also focused more on whole family participation and increasing family physical activity time versus decreasing sedentary behavior for TLD. Epstein and colleagues (2001) provided a detailed description of implementation of the TLD and indicated a red food goal and an equivalent caloric goal. We chose to focus on the “red” equivalents. In addition, their general activity program described a lifestyle physical activity recommendation which was to accumulate at least 30 min of MPA per day and increase goals from there. With the introduction of objective measures of PA, we chose to use an equivalent recommendation with an objective monitor—steps per day.

**Table 1:** Key program components in Epstein’s Traffic Light Diet and Building Healthy Families

	Program components	Epstein and colleagues’ key TLD components	Adaptation of key components in BHF
**Contextual**	**Delivery team**	Behavioral therapist	*BHF components developed by:* community dietitian, clinical behavioral psychologist, and academic exercise scientist
	**Format/setting**	Face-to-face at University Medical Center	Face-to-face at rural comprehensive undergraduate university
	**Length**	60 min for 8–16 weeks followed by monthly treatment to 6 months	90 min for 12 weeks followed by 60 min for 4 refresher sessions up to 6 months
**Population**	**Participants**	Children 6–12 years of age with a BMI ≥ 95th percentile	Children 6–12 years of age with a BMI ≥ 95th percentile
	**Family based**	Participating parent and child must attend and participate	Participating parent and child must attend and participate
	**Group treatment**	Some cohorts were parent and child separately and some were parent and child together	Parent and child together
**Major intervention principles**	**Core curriculum components**	Behavioral principles related to eating and physical activity patterns, role playing, and problem solving	Behavioral principles related to eating and physical activity patterns, role playing, and problem solving
	**Goal setting**		
	*Diet*	Elimination of “red” foods each week until eating 2 servings of red foods per day Energy intake target range: 1000–1500 kcal/day	Elimination of “red” foods each week until eating 2 servings of red foods per day
	*Physical activity*	Lifestyle goal of 30 min/day of moderate-to-vigorous physical activity (MVPA) or 180 min/week self-reported	Increased baseline steps by 1000–2000 steps/day and required meeting goal 5 of 7-day accelerometer
	*Weight loss*	Children 0.5–1 lb/week; adults 1–2 lbs/week	Children 0.5–1 lb/week; adults 1–2 lb/week
	**Nutrition education**	Traffic Light Diet: identify foods as red, yellow, and green based on fat, sugar, and calories. Red foods > 5 g fat per serving, low nutrient density. Yellow foods between 2 and 4.9 g fat, green foods 0–1.9 g fat.	Traffic Light Eating Plan: identify foods as red, yellow, and green based on fat, sugar, and calories. Red foods > 5 g fat or 200 kcal per serving, low nutrient density. Yellow foods between 2 and 4.9 g fat, green foods 0–1.9 g fat.
	**Behavioral skills**		
	*Self-monitoring*	Record daily eating and physical activities in habit books—provide feedback and reinforcement techniques	Record daily eating and physical activities in habit books—provide feedback and reinforcement techniques
	*Rewards/reinforcements*	Identifying opportunities for praise and changing rewards to nonfood based	Identifying opportunities for praise and changing rewards to nonfood based
	*Role modeling*	Parent modeling healthy behaviors	Parent modeling healthy behaviors
	*Stimulus control/modification of environment*	Environmental changes to reduce access to “red” foods, shop differently, cook healthier, decrease sedentary time	Environmental changes to reduce access to “red” foods, shop differently, cook healthier, decrease sedentary time, increase family activity time
	**Physical activity**	Lifetime activities promoted and sedentary time discouraged, weekly goals—20 min on 6 days per week	During weekly sessions parents and children engage in 20–40 min of moderate activities together. Weekly goals are given using step counters and family activities encourage with local resources.

All modifications reported were planned/proactive; therefore, the term adaptations will be used when referring to specific modifications made for BHF. Content changes for BHF occurred at the cohort level for each cohort; that is, content of the program was not modified for a specific participant within a cohort*.* The core elements of the original TLD were preserved for the initial adaptation for cohort 1 (and all subsequent cohorts). Finally, there was no attempt to change how the core elements functioned within the intervention. Table 2 provides a heat map of modifications by FRAME constructs and coding categories by cohort.

### BHF Program Adaptations Made During Implementation

The implementation team and program coordinator made adaptations during implementation, including scale-up for, and implementation within, the remaining 8 cohorts (Table 3). Results are described below according to the remaining categories within FRAME: what is modified (i.e., content, context, training and evaluation, and implementation and scale-up activities), type or nature of context and content-level modifications, and the reasons for the modification, including (a) the intent or goal of the modification and (b) contextual factors that influenced the decision.

**Table 2:**
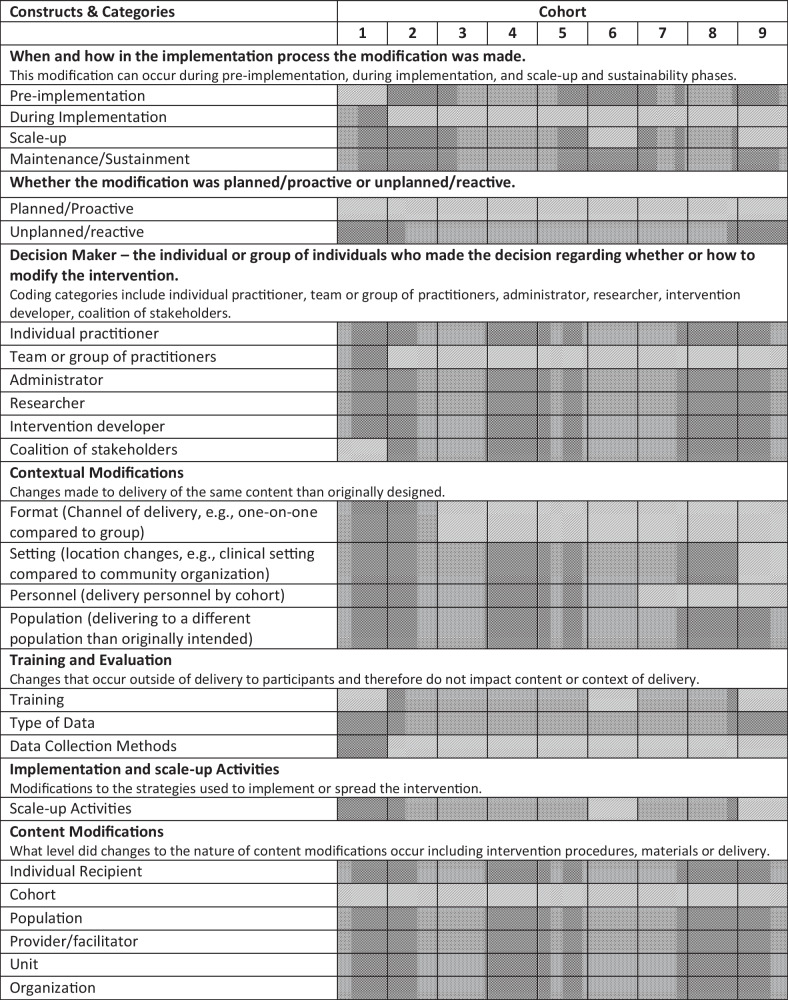

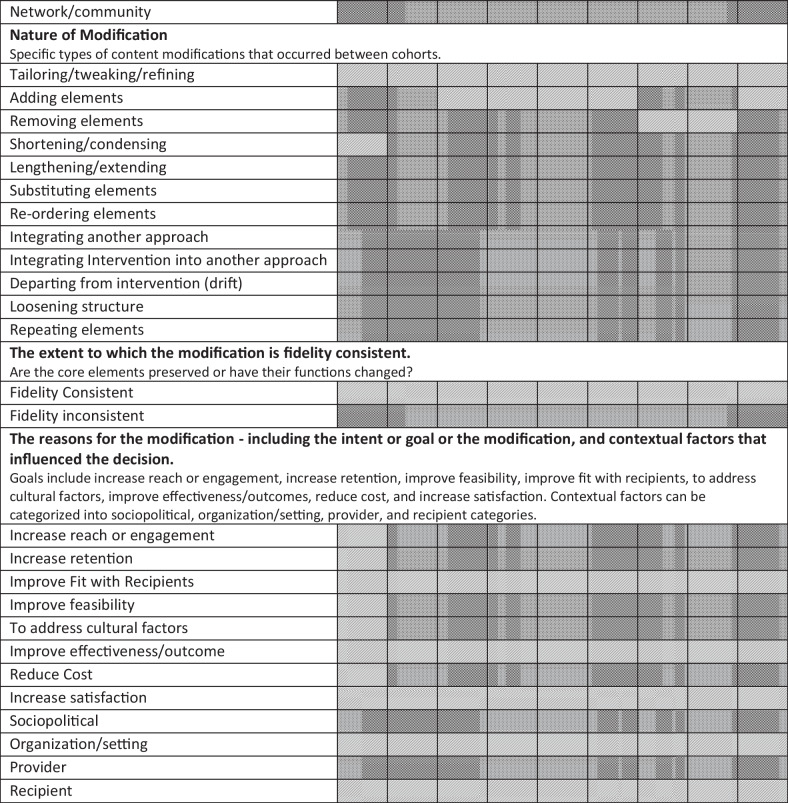
Adaptations by FRAME constructs and coding categories

Cells shaded in light gray indicate that any adaptation was made within that reporting code. Cells shaded in dark gray indicate that no adaptation was made within that reporting code

**Table 3:**
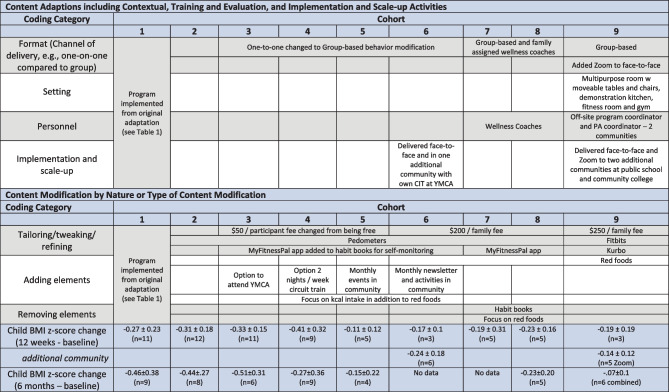
Adaptations during implementation after cohort 1

Blank cells reflect an area where no modifications were made after cohorts 1 and 2. See Table 1 for all initial adaptations

#### What Is Modified

BHF was implemented in a face-to-face format for all cohorts. There were no format changes for the delivery of the nutrition education and physical activity components. For cohorts 1 and 2, lifestyle modification included one-on-one behavioral modification meetings with each family and the behavioral psychologist. Starting in cohort 3, group behavioral modification strategies were used.

#### Nature or Type of Content Modification

Behavior modification sessions began with one-on-one meetings with the BHF coordinator before or after the nutrition education session. This was logistically difficult and extended sessions considerably. It was noted by the implementation team that most of the sessions were similar in content and that successful families could share their tips with other families in group-based modification sessions in lieu of one-on-one meetings. Subsequently, the BHF lifestyle modification coordinator felt she was not able to make personal connections with all families during group-based sessions. Therefore, the concept of wellness coaching was implemented during cohorts 7 and 8 to provide one-on-one contact with each family every week. Existing BHF personnel provided wellness coaching to participating families and developed rapport when families arrived for each weekly session. Personnel greeted families each week and initiated conversations with participants and family members about self-monitoring, goal attainment, and overcoming barriers over the past week. Wellness coaches shared this information with the lifestyle modification coordinator to inform the delivery of the lifestyle modification component of each session. Smaller participation in cohort 9 (*n* = 3 families) lead to the elimination of wellness coaches. With smaller participation numbers, the lifestyle modification coordinator was able to talk to each family individually throughout the sessions (Table 3).

#### Setting

The setting for BHF remained the same for the first 8 cohorts. Cohort 9 was delivered in a new facility on the university campus. This new facility included a multipurpose room with moveable tables and chairs and a demonstration kitchen. The population served across the 9 cohorts included children aged 6–12 years old with a BMI percentile 95 or greater (and their family members).

#### Implementation for Scale-Up Activities

Implementation for scale-up activities took place in cohorts 6 and 9. Cohort 6 consisted of two implementation groups. One group was implemented in the host university classroom, and the second group was implemented in a community of about 5000 people approximately 30 mi from the university. The additional implementation group within cohort 6 was delivered at a local YMCA by an independent community implementation team. An online resource was created to share the same BHF program and materials. The community implementers were provided technical assistance and support from the original implementation team. The original implementation team worked weekly with the additional implementation team to ensure understanding of materials and program fidelity. A graduate student from the university attended every session to conduct direct observations of program fidelity.

In addition to cohort 9 being delivered face-to-face in the new host university facility, it was implemented synchronously via Zoom in two additional community settings (population ~ 3359–7698) within 65 to 100 mi from the university at a local high school and a community college. Thus, there were three groups: one face-to-face with the implementation team at the university and two remote groups each with a local program coordinator and physical activity coordinator. The program coordinator at each site was there to greet the families, conduct weigh ins, hand-out and collect habit books, log physical activity, and secure necessary supplies for the weekly education sessions. The physical activity coordinator at each site was responsible for delivering the physical activity component to participants and their family members. The nutrition education and lifestyle modification components were delivered via Zoom by the original university implementation team members. For both cohorts 6 and 9, training was provided to the remote implementation teams in the form of a free BHF website that provided access to course materials and instructional videos, as well as a weekly telephone meeting (Table 3). However, the execution of the multiple groups via Zoom and in person by the original implementation team proved challenging, and at times, attention in any one location was impacted.

#### Content Modification by Nature or Type of Content Modification

##### Tailoring/Tweaking/Refining

Tailoring, tweaking, or refining is defined as any minor change to the intervention that leaves all major intervention principles and techniques in place. In a world of continually changing technology and external influences on children, it is important to modify program components not just to avoid becoming obsolete (Riley et al., 2015), but to stay up with current trends while maintaining the core principles of the program. Epstein and colleagues TLD used self-reported physical activity where BHF used accelerometers in cohort 1. The choice to use accelerometers was to obtain objectively measured physical activity data. When implemented, participant feedback was unfavorable to using accelerometers due to the inability to see their real-time activity progress which decreased their motivation to use the device or increase activity. Thus, the implementation team transitioned to pedometers to measure physical activity, and as technology changed, wearable activity monitors worn on the wrist for cohorts 2–9 (Table 3). Making the change to wearable activity monitors decreased the number of lost devices and were preferred by participants. Although the wearable devices were preferred by participants, the implementation team faced challenges unique to rural areas with collecting data (e.g., internet connections, computers with downloading/Bluetooth capabilities). Ultimately, the implementation team decided to have participants record their steps per day within in their habit books.

Changes in how participants self-monitored lifestyle modifications was also influenced by emerging health-related apps and physical activity measuring devices due to participants being aware of these newer technology products and wanting to use them. Cohort 1–3 participants used pencil and paper habit books. Cohort 4–6 participants continued to use habit books in addition to using MyFitnessPal (MyFitnessPal, Inc., Austin, TX), the electronic web-based application (app) available via computer and or mobile device. MyFitnessPal was used exclusively by participants in cohorts 7 and 8. Cohort 9 participants used a different child-focused app, Kurbo (Kurbo, Inc., San Francisco, CA), that monitored “red foods.” BHF personnel also made changes to the cost of the BHF program. Costs ranged from free to $50 per qualifying participant and parent to charging by family (e.g., $200 for a family of ≤ 4 participants or $250 for a family of ≥ 5 participants) based on a sliding scale (Table 3). Further, when BHF participants were required to record food consumption and physical activities performed in a hand-written habit book, parents needed to interact with their child and discuss foods and activities to help the child to fill in their habit book. Although parents appreciated the apps as recording tools, recording by child participants, the primary participant, was greatly reduced when using technology facilitated approaches.

##### Adding Elements

Additional activities or materials consistent with the core principles of BHF were added. These additions occurred both during the weekly sessions and outside the weekly sessions (Table 3). In addition to red food consumption, cohorts 4–6 incorporated a focus on calorie intake with the continued use of MyFitnessPal. A sole focus on calorie intake for behavior modification using MyFitnessPal took place during cohorts 7 and 8. The program refocused behavior modification to red foods in cohort 9. The nutrition-related program goal of reducing red foods remained in place for all cohorts even with increased focus on calorie intake for nutrition-related behavior modification for cohorts 4–8. Cohorts 3–6 included introducing families to facilities and programs for physical activity promotion outside of the weekly scheduled session (Table 3).

##### Individual Outcomes

Ninety qualifying children across 84 families initiated participation, 75 children completed 12-week assessments (83.3% retention), and 47 returned for 6-month assessments (52.2% retention from baseline). Children that completed the 12-week program attended 85.5 ± 17.3% of the education sessions compared to 26.3 ± 22.1% attendance among those that did not complete the 12-week assessments (*p* < 0.05). Results of a *t* test indicated no significant difference (*p* ≥ 0.05) at baseline between participants that completed the 12-week program and those that did not complete the program. Results indicated that the BMI *z* score in children was reduced by 0.31 ± 0.17 at 6 months. Table 3 illustrates the changes in BMI *z* score at 12 weeks and 6 months for each cohort.

## Discussion

This paper fills the current gap in the literature related to evaluating modifications to evidence-based interventions that could result in differences in program effectiveness (Chambers & Norton, 2016). The BHF adaptation based on Epstein and colleagues (2001) TLD provided a PWMI for families in micropolitan or rural communities where access to medical centers or large hospital programs is not available. Our project resulted in a number of generalizations that can be made related to categorizing the type and timing of adaptations, the potential importance of an interdisciplinary implementation team, and the potential decline in effectiveness of an adapted PWMI over time. We were also able to consider methods for assessing adaptations and modifications based on current recommendations (Stirman et al., 2019). Finally, the project helped to identify PWMI implementation factors that were relatively stable over time (i.e., needing little adaptation) and areas where flexibility could be provided to community implementation teams.

Using the FRAME (Stirman et al., 2019) reporting structure, the changes that were documented were (1) planned and occurred pre-implementation, (2) based on decisions from local stakeholders (e.g., school administrator, members of the implementation team), and (3) specific to changes in content and context—with a focus on implementation and potential for local scale-up. The nature of the adaptations included adding elements (whole of family approach), removing elements (calorie counting), and substituting elements (steps for minutes of physical activity). While there are no published articles outlining the core elements of the TLD, previous publications focused on social cognitive theory and self-regulation strategies (Epstein et al., 2001) in addition to including components related to lifestyle modification, healthful eating, and physical activity—all core components of BHF—suggesting a strong preservation of core elements.

Other PWMIs implemented in rural communities have used similar program structures (e.g., number and duration of sessions; similar core elements) and resulted in significant reductions in BMI *z* scores or percentile rankings (Hill et al., 2019; Janicke, 2013; Janicke et al., 2008a, 2009). However, the magnitudes of effect in other rural PWMI studies appear to be smaller than those achieved in BHF (and the TLD). For example, Project Story (Janicke et al., 2008b), a PWMI adapted from the TLD delivered by cooperative extension health educators, and iChoose (Hill et al., 2019), a PWMI adapted from Bright Bodies (Savoye et al., 2007) delivered by Parks and Recreation staff, resulted in BMI *z* score reductions at program completion between 0.05 and 0.14 (Hill et al., 2019; Janicke et al., 2008a). In comparison, results from BHF indicated a reduction in BMI *z* score by 0.31 ± 0.17 at 6 months exceeding the recommended reduction of ≥ 0.25 in order to improve cardiometabolic risks in adolescents (Ford et al., 2010). This study contributes to the proposition that PWMIs in micropolitan and rural communities may be most effective when implemented by an interdisciplinary team—even when members of the team are not housed within the same organization.

While reductions in BMI *z* score for each of the 9 cohorts were at a level that has been shown to achieve clinical outcomes (Ford et al., 2010; US Preventive Task Force et al., 2017), the BMI *z* score reductions for cohorts 5–9 were not as large as the changes in cohorts 1–4, which calls program drift into question. Program drift (Chambers & Norton, 2016) is the assumption that program outcomes may be attenuated over time. One potential cause of this attenuation is that implementation drift (i.e., reduced fidelity to protocol) may have occurred in later cohorts. Cohorts 5 and 6 focused more on calorie intake than red food intake, and possible changes in the ways that tracking was completed in cohorts 7 and 8 using technological approaches in place of habit book use could be one area of implementation drift. Focusing on calories and online food trackers may have facilitated parent self-monitoring; however, child self-monitoring decreased and was often completed by the parent instead of the child. Cohorts 5–9 had a smaller number of participants than cohorts 1–4. It is possible that the small number of participants in each cohort influenced the group dynamics in a way that impacted outcomes. However, as a retrospective study, we do not have measurements or insight into the potential reasons. The cost of the program changed from free to $50 per participant on a sliding scale to require “buy-in” from families. We then changed to cost per family rather than individual and did not receive any concern from families. If families discontinued participation, we provided a partial refund of the program. Further research is needed in this area to determine if variability in cohort outcomes was due to program factors or, potentially, due to participant or other contextual factors.

When considering the adaptations above, it also appears that if the core elements of BHF are maintained, there is potential for flexibility in the strategies in which they are applied (e.g., form of self-monitoring, reducing red foods with or without calorie counting, or delivered to parent–child dyads or to whole families). Future research on, and practice of, PWMI implementation in micropolitan or rural areas would benefit from resources that support communities in the consistent use of core elements while also providing some flexibility in how those core elements can be applied. In micropolitan and rural areas, food deserts, lack of community-based nutrition education, and food insecurities are all potential issues that must be considered. For example, a grocery store tour in a rural area in a food desert will look completely different than an urban grocery store. Modifying recipes for rural residents may be different based on availability of food substitutes.

To scale BHF and increase reach, different approaches were piloted in cohorts 6 and 9. In cohort 6, a community implementation team had online access to BHF program materials paired with weekly 1:1 meetings and technical assistance from the original implementation team. Implementation fidelity measured by direct observation was high, and BMI *z* score reductions in this group matched or exceeded other cohorts. Although the program was successful, the amount of resources necessary to implement BHF and the number of qualifying children limited the number of times the program could be implemented. In cohort 9, we tried to mitigate the intensity of community-based resources (e.g., a full local implementation team) with a remote led option. We identified two rural communities, and instead of identifying local implementation teams, we asked them to have an on-site program coordinator and physical activity coordinator. The on-site program coordinator was responsible for recruiting participants and providing on-site assistance to families.

Having three groups simultaneously implement BHF, one face-to-face and two via Zoom, posed challenges with the university implementation team capacity, and the BMI *z* score reduction for the groups over Zoom was lower than the face-to-face group. The BMI *z* score reduction for all cohort 9 participants was lower than all other cohorts at 6 months. It was difficult for the implementation team to provide attention to both the participants in the room and to two communities over Zoom. Trying to engage children in both settings and minimize distractions of people talking over each other were difficult for discussions. Sessions may have been implemented better if working directly with families over Zoom. This was a retrospective review of BHF adaptations, and therefore, we are unable to make causal inferences about variability in outcomes. Additional strategies for scale-up need to be explored to systematically test and identify the approach that will retain effectiveness while recognizing limitations on resources within local communities.

The application of the FRAME approach to document adaptations from a retrospective perspective was not ideal. However, our experience using the approach indicated that it may be possible to use a more pragmatic approach to tracking adaptations. Specifically, all adaptations proposed across the 9 cohorts were completed with the goal to retain the core focus on physical activity, nutrition, and lifestyle modification. Further, adaptations also aligned with the underlying behavioral theory (i.e., social cognitive theory) principles and proposed hypotheses (e.g., use of whole of family participation to address environmental influences on the individual and behavior). While the specificity of the FRAME approach across the when, who, what, why, level, context, reason for, and nature of adaptations provides an exceptional variety to describe very nuanced adaptations, the focus on underlying theoretical or mechanistic principles is relatively underdeveloped. Iterative adaptations to BHF were completed to improve the fit of BHF to the setting and participants and have contributed to a sustained community PWMI that adheres to the underlying principles and core elements of other evidence-based PWMIs. As future PWMI work continues, one goal could be towards parsimony of adaptation tracking based on factors that are most likely to be related to sustained effectiveness or effectiveness when taken to scale. Monitoring adaptations and related changes to outcomes can play a role in long-term sustainability and effectiveness.

### Strengths

This paper provides an examination of real-world adaptation that resulted in BHF, a sustainable and effective approach to reducing childhood obesity in a medically underserved area. Data that were reported in this project were primarily drawn from existing program implementation data that was tracked in real time as the intervention was being delivered. As a result, the analysis of these data based on the FRAME approach likely captured all primary changes made to improve the fit of BHF within the local implementation context.

### Limitations

While the existence of recorded data on adaptations is a strength, the use of a retrospective approach has limitations such as a reliance on qualitative adaptation data that was not initially intended to provide a detailed accounting of PWMI changes. This suggests that potential reasons for adaptations and the degree to which adaptations were consistently applied relies on implementation team recall. Some protection against recall bias and therefore reducing the likelihood that major adaptations were missed include the consistency of the core implementation team members across all 9 cohorts. This team also met regularly before, during, and after each cohort to discuss potential modifications. In addition, as a community-placed program, BHF participating families did not return for follow-up assessments to the same degree as participants who completed follow-up within the context of a research study. As such, weight status changes may be inflated due to attrition of families that may not have maintained weight status changes.

## Conclusion

Our findings suggest that an implementation team—including professionals across community organizations—can co-lead a program like BHF and achieve similar outcomes to previous efficacy trials. This is consistent with other programs that have been developed for implementation by health educators in community settings which have also acheived BMI *z* score reductions (0.17) in the range necessary to improve health outcomes (Perry et al., 2017).

## Data Availability

Data available on request by contacting the corresponding author.
